# Direct Involvement of Cranial Nerve V at Diagnosis in Patients With Diffuse Intrinsic Pontine Glioma: A Potential Magnetic Resonance Predictor of Short-Term Survival

**DOI:** 10.3389/fonc.2019.00204

**Published:** 2019-04-04

**Authors:** Giovanna Stefania Colafati, Ioan Paul Voicu, Chiara Carducci, Massimo Caulo, Maria Vinci, Francesca Diomedi-Camassei, Pietro Merli, Andrea Carai, Evelina Miele, Antonella Cacchione, Paolo Tomà, Franco Locatelli, Angela Mastronuzzi

**Affiliations:** ^1^Department of Imaging, Neuroradiology Unit, Bambino Gesù Children's Hospital, IRCCS, Rome, Italy; ^2^Department of Onco-haematology, Cell and Gene Therapy, Bambino Gesù Children's Hospital, IRCCS, Rome, Italy; ^3^Department of Neuroscience and Imaging, ITAB-Institute of Advanced Biomedical Technologies, University G. d'Annunzio, Chieti, Italy; ^4^Pathology Unit, Department of Laboratories, Bambino Gesù Children's Hospital, IRCCS, Rome, Italy; ^5^Neurosurgery Unit, Department of Neuroscience and Neurorehabilitation, Bambino Gesù Children's Hospital, IRCCS, Rome, Italy; ^6^Radiology Unit, Department of Imaging, Bambino Gesù Children's Hospital, IRCCS, Rome, Italy

**Keywords:** child, MRI, DIPG = diffuse intrinsic pontine glioma, cranial nerves, biomarkers

## Abstract

**Background:** Diffuse intrinsic pontine glioma (DIPG) has a dismal prognosis. Magnetic resonance imaging (MRI) remains the gold standard for non-invasive DIPG diagnosis. MRI features have been tested as surrogate biomarkers. We investigated the direct involvement of cranial nerve V (CN V) in DIPG at diagnosis and its utility as predictor of poor overall survival.

**Materials and Methods:** We examined MRI scans of 35 consecutive patients with radiological diagnosis of DIPG. Direct involvement of CN V was assessed on the diagnostic scans. Differences in overall survival (OS) and time to progression (TTP) were analyzed for involvement of CN V, sex, age, tumor size, ring enhancement, and treatment regimen. Correlations between involvement of CN V and disease dissemination, magnet strength and slice thickness were analyzed. Statistical analyses included Kaplan-Meier curves, log-rank test and Spearman's Rho.

**Results:** After excluding six long-term survivors, 29 patients were examined (15 M, 14 F). Four patients presented direct involvement of CN V. Histological data were available in 12 patients. Median OS was 11 months (range 3–23 months). Significant differences in OS were found for direct involvement of CN V (median OS: 7 months, 95% CI 1.1–12.9 months for involvement of CN V vs. 13 months, 95% CI 10.2–15.7 for lack of involvement of CN V, respectively, *p* < 0.049). Significant differences in TTP were found for the two treatment regimens (median TTP: 4 months, 95% CI 2.6–5.3 vs. 7 months, 95% CI 5.9–8.1, respectively, *p* < 0.027). No significant correlation was found between involvement of CN V and magnet strength or slice thickness (*r* = −0.201; *p* = NS). A trend toward positive correlation was found between direct involvement of CN V at diagnosis and dissemination of disease at follow-up (*r* = 0.347; *p* < 0.065).

**Conclusions:** In our cohort, direct involvement of CN V correlated with poor prognosis. Based on our data, we suggest that in DIPG direct involvement of CN V should be routinely evaluated on diagnostic scans.

## Introduction

Diffuse Intrinsic Pontine Glioma (DIPG) remains the pediatric brain tumor with the worst prognosis (1). Despite recent advances in detailing the biology of DIPG (2), <5% of affected children survive at 3 years irrespective of histological grading and median overall survival (OS) from diagnosis is 9–12 months (3).

The most common clinical features include cranial nerve palsy, ataxia, and long tract signs.

MRI is the gold standard non-invasive method for DIPG diagnosis (4, 5) based on both major and minor diagnostic criteria (6–8) in the context of a typical clinical presentation. Typical MRI findings consist of a large expansive pontine lesion that is hypointense or isointense on T1 weighted (T1w) imaging, hyperintense on T2 weighted (T2w) and fluid-attenuated inversion recovery (FLAIR) imaging, and of variable enhancement with gadolinium contrast agent (4).

Conventional and advanced MRI parameters (3, 9–16) have been tested in different studies in an effort to predict survival at the time of diagnosis. Ring contrast enhancement has been suggested to inversely correlate to OS (17).

Direct cranial nerve involvement in brainstem gliomas has been suggested to represent an extra-axial extension of the tumor. This feature seems to be related to glial cells extending from cranial nerve nuclei to the root entry zone (18–20). Cranial nerves V, VII and VIII have been described to be predominantly involved, but no correlation to tumor grade has been demonstrated.

In the present study we describe the direct involvement of CN V at diagnosis in patients with DIPG in a consecutive single institution series and correlate this imaging feature with clinical variables to test its value as prognostic biomarker.

## Materials and Methods

### Patients

The Institutional Review Board approved this research waiving the need for informed consent from patients or their parents/legal guardians for this specific retrospective analysis. The study was conducted in agreement with the principles contained in the Declaration of Helsinki.

We collected data from 35 consecutive patients diagnosed with DIPG in our Institution, who underwent MRI from 1st January 2003 to 1st February 2018. Diagnosis was based on clinical and radiological criteria consistent with DIPG (6, 7).

Patients with either, radiological findings inconsistent with DIPG, and/or a diagnosis of neurofibromatosis, and/or diagnostic exams which did not include the entire neuraxis were excluded. Patients with an overall survival longer than 24 months were not included in the survival analysis, although their characteristics were reported in detail.

### Magnetic Resonance Imaging

MR examinations were performed on a 3 Tesla (3 T) magnet (Siemens Magnetom Skyra, Erlangen, Germany) or on a 1.5 Tesla (1.5 T) magnet (Siemens Magnetom Vision Plus, Erlangen, Germany). All patients underwent at least T1w sagittal, T1w and T2w axial sequences, FLAIR, T2w coronal and diffusion (DWI) sequences. These sequences were used for image analysis. Two different acquisition protocols were implemented on the two scanners. Slice thickness of the acquired sequences was 4 mm on the 1.5 T magnet and 3 mm on the 3 T magnet. All patients received scan of the entire neuraxis. A contrast medium agent was used in all studies. Patients deemed unable to cooperate for age-related and/or clinical status-related reasons were imaged under general anesthesia.

For each patient, radiological and clinical data were collected. In order to reduce the possibility of an interpretation bias, patient scans were first anonymized and then transferred to a separate workstation.

Two expert neuroradiologists from different institutions, both with 20 years of experience, reviewed patient scans in consensus to collect radiological data. This method was used for all MRI analyses unless otherwise specified.

The radiological criteria for DIPG diagnosis (T1 hypointense and T2 hyperintense lesions with poorly defined margins involving more than 50% of the pons) were first assessed.

**Figure 1:**
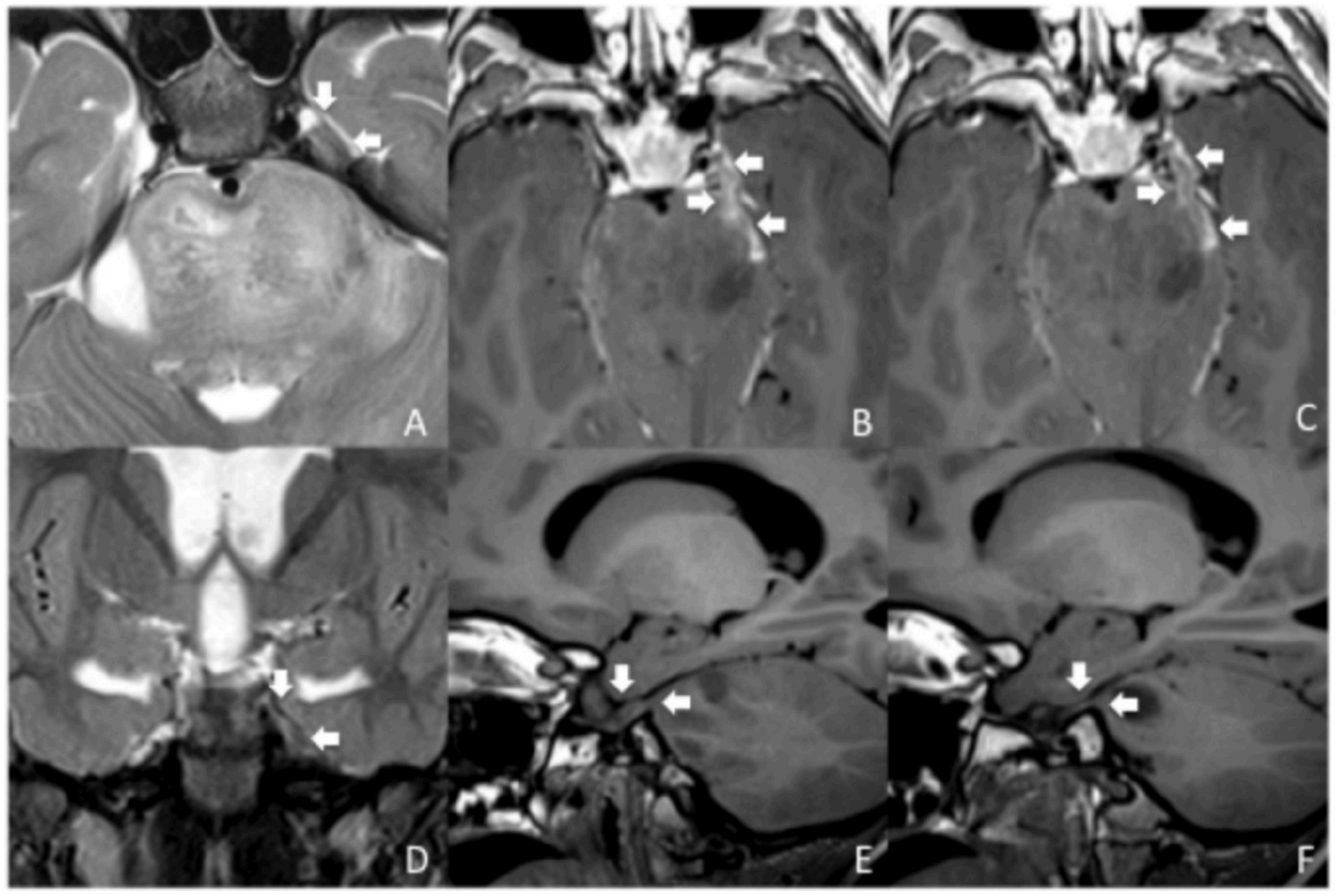
MRI diagnosis of direct involvement of CN V by DIPG in a 4-years-old boy. Axial TSE T2 **(A)**, para-axial reformatted T1 post-contrast **(B,C)**, coronal TSE T2 **(D)** and para-sagittal reformatted T1 images **(E,F)** show thickening of the left CN V causing filling of the left Meckel cave, consistent with direct cranial nerve involvement from the tumor (**A–E**, white arrows). The pathologic thickening of the left CN V is associated with contrast enhancement (**B,C**, white arrows). Pathologic thickening of CN V is better delineated when comparing it with the contralateral normal V cranial nerve (**F**, arrows).

Diagnostic scans were then reviewed to investigate direct involvement of CN V by the tumor. Direct involvement of CN V was defined by the thickening and/or abnormal signal intensity involving the root entry zone and the proximal cisternal course of the cranial nerve, contiguous with the tumor, visible on at least two sequences (Figure 1). The absence of contrast enhancement did not exclude the diagnosis of involvement of CN V. Exclusion of leptomeningeal spread was deemed necessary to confirm the diagnosis of direct involvement of CN V. Leptomeningeal spread was defined as the region of pathologic contrast enhancement consistent with disease localization confined in any region of the neuraxis and not contiguous with the tumor.

In order to investigate if in our cohort other MRI features could affect patient survival we collected other two known potential MRI surrogate biomarkers: tumor size and ring enhancement.

Tumor size was defined as the product of the longest perpendicular diameters of the tumor, measured on an axial plane on FLAIR sequences. This method has been evaluated to assess tumor response to therapy (21) and has been debated in literature (22). The presence of ring enhancement was assessed on T1 post-contrast images.

For each patient, all follow-up scans were evaluated for the presence of involvement of CN V and dissemination of the disease. All patients underwent a fixed follow-up protocol. Controls at follow-up were performed 30 days after radiotherapy, and thereafter every 3 months, unless new symptoms occurred.

Since both a 1.5 Tesla magnet and a 3 Tesla magnet were employed in our study with two different protocols, potential correlations between the involvement of CN V with the magnet strength and with slice thickness were also analyzed.

An experienced neuro-oncologist blinded to image analysis collected clinical data. The neuroradiologists were blinded to the results of clinical data collection.

### Statistical Analysis

Quantitative variables were reported as median value and ranges, while categorical variables were expressed as absolute values and percentages.

Overall survival (OS) was defined as the length of time from the date of diagnosis to the date of death or last follow-up. Time to progression (TTP) was defined as the length of time from the date of diagnosis until disease progression. Tumor progression was defined as radiological progression, associated with worsening of pre-existing symptoms or the appearance of new symptoms. Radiological progression was defined as an increase >25% in tumor size, based on the RAPNO criteria (21), and/or metastatic progression of the disease. Metastatic progression was defined as new distant signal alterations and/or leptomeningeal enhancement, consistent with disease. Probability of OS and TTP were calculated according to the Kaplan and Meier method (23) and expressed as median OS and median TTP, respectively. All results were expressed as probability or cumulative incidence (%) and 95% confidence interval (95% CI).

Differences in OS and TTP were estimated with the log–rank test (Mantel–Cox). Direct cranial nerve involvement at diagnosis, the presence of ring enhancement and sex were used as categorical variables. Tumor size and patient age were stratified for values superior and inferior to their median values.

Spearman's rho was used to evaluate the relationship between the involvement of CN V at diagnosis with the magnet strength, and the slice thickness, as well as with the tumor dissemination at follow-up.

*P*-values < 0.05 were considered to be statistically significant; *p*-values between 0.05 and 0.1 were not considered statistically significant but were reported in detail in the text; *p*-values ≥0.1 were reported as non-significant (NS).

Statistical analysis was performed using SPSS statistics (version 20). Survival analysis used 31/08/2018 as reference date.

## Results

For this study, we initially considered 35 patients radiologically diagnosed with DIPG. Six patients presented an OS of more than 24 months and were not included in the survival analysis, but their characteristics were reported in detail. Clinical, radiological and histological characteristics of long-term survivors are shown in Table 1.

**Table 1:** Clinical, radiological, and available histological characteristics in long-term survivors with DIPG from our cohort.

**Patient ID**	**Sex**	**V cranial nerve involvement**	**Status**	**Tumor size**	**Ring enhancement**	**Treatment**	**Histological diagnosis**	**Age at diagnosis**	**Progression**	**Time to progression**	**Dissemination at follow up**	**Overall survival**
8	F	Yes	DOD	2,035	No	B	Low grade astrocytoma, H3K27 wild type	104	Yes	7	Yes	32
15	M	No	DOD	1,564	No	B	Diffuse midline glioma, H3K27 mutant	79	Yes	9	No	27
16	M	No	AWD	2,070	Yes	B	Pilocytic astrocytoma	24	Yes	63	No	68
24	M	No	AWD	2,296	No	A	Not available	45	No	183	No	183
26	F	No	DOD	1,692	No	B	Diffuse midline glioma, H3K27 mutant	46	Yes	13	No	25
28	M	No	DOD	1,800	No	A	Not available	42	Yes	6	No	31

*ID, patient number; sex, male (M) or female (F); V cranial nerve involvement, direct V cranial nerve involvement at diagnosis present (yes) or absent (no); Status, patient outcome including alive with disease (AWD), dead of disease (DOD) or lost at follow-up (LAF); Tumor size, tumor size expressed in mm^2^; Ring enhancement, ring enhancement present (yes) or absent (no) at diagnosis; Treatment, patient first line of treatment with radiotherapy and Temozolomide (A) or with radiotherapy, Nimotuzumab and Vinorelbine (B); Histological diagnosis, available histological diagnosis, according to the WHO 2016 classification; Age at diagnosis, age at diagnosis expressed in months; Progression, the presence (yes) or absence (no) of clinical and/or radiological signs of disease progression; Time to progression, the length of time from the date of diagnosis of DIPG until the radiological and/or clinical progression of the disease, expressed in months; Dissemination at follow-up, distant localization of the tumor present (yes) or absent (no) on the follow-up scans; Overall survival, overall survival expressed in months*.

The final cohort consisted of 29 patients: clinical, radiological and histological data are shown in Table 2. Among them, there were 15 boys (M = 51.7%) and 14 girls (F = 48.3%). Median age at diagnosis was 5.7 years (range 2.5–14.4). Histological confirmation was also obtained in a subgroup of 12 patients, as since December 2015 robot-assisted trans-frontal stereotactic needle biopsy has routinely been proposed at diagnosis to patients with DIPG referred to our Institution (24). All patients undergoing histological confirmation presented a H3K27M mutation. Before 2011, all patients received first-line radiation and chemotherapy according to the Stupp protocol (25). After 2011, all patients received first-line treatment with radiation, Nimotuzumab and Vinorelbine (26).

**Table 2:** Clinical, radiological, and histological characteristics of the patients with DIPG from our cohort.

**Patient ID**	**Sex**	**Status**	**Age at diagnosis**	**Treatment**	**Available histology**	**Histological diagnosis**	**Time to progression**	**Overall survival**	**CN V diagnosis**	**CN V follow-up**	**Tumor size**	**Ring enhancement**	**Dissemination at follow up**	**Scanner**	**Slice thickness**
1	M	DOD	81	B	No	n/a	14	18	No	no	2,000	No	Yes	3 T	3 mm
2	M	DOD	54	A	No	n/a	2	4	Yes	Yes	1,768	No	Yes	3 T	3 mm
3	M	DOD	100	A	No	n/a	3	5	No	No	1,116	No	No	1.5 T	4 mm
4	F	DOD	57	A	No	n/a	3	10	No	No	851	No	No	1.5 T	4 mm
5	F	DOD	112	B	Yes	Diffuse midline glioma, H3K27M mutant	11	20	No	No	1,845	Yes	No	3 T	3 mm
6	M	DOD	51	A	No	n/a	3	7	Yes	No	1,925	No	No	1.5 T	4 mm
7	M	DOD	154	B	Yes	Diffuse midline glioma, H3K27M mutant	7	8	No	No	999	No	No	3 T	3 mm
9	M	DOD	61	B	No	n/a	9	13	No	No	1,344	No	No	3 T	3 mm
10	F	LAF	166	B	Yes	Diffuse midline glioma, H3K27M mutant	6	6	No	No	1,000	No	No	3 T	3 mm
11	F	DOD	49	B	No	n/a	7	14	No	No	1,739	No	No	3 T	3 mm
12	F	DOD	50	A	No	n/a	7	13	No	No	1,344	No	No	1.5 T	4 mm
13	M	DOD	76	A	No	n/a	8	10	No	No	1,462	Yes	No	1.5 T	4 mm
14	M	DOD	51	B	No	n/a	5	8	No	No	1,152	No	Yes	3 T	3 mm
17	M	DOD	81	A	No	n/a	7	16	No	No	2,552	No	Yes	1.5 T	4 mm
18	F	DOD	46	B	No	n/a	3	7	No	No	2,160	No	No	3 T	3 mm
19	F	DOD	36	A	No	n/a	4	10	Yes	Yes	1,764	Yes	No	1.5 T	4 mm
20	F	DOD	49	B	No	n/a	2	3	No	No	1,161	No	No	3 T	3 mm
21	M	DOD	72	B	No	n/a	8	7	No	No	2,262	Yes	No	3 T	3 mm
22	F	DOD	81	B	Yes	Diffuse midline glioma, H3K27M mutant	7	9	No	No	850	No	No	3 T	3 mm
23	F	DOD	51	A	No	n/a	4	23	No	No	1,800	Yes	No	1.5 T	4 mm
25	M	DOD	173	B	No	n/a	8	12	No	No	1,364	No	No	3 T	3 mm
27	M	DOD	67	B	Yes	Diffuse midline glioma, H3K27M mutant	8	12	Yes	No	1,824	No	Yes	3 T	3 mm
29	F	DOD	68	B	Yes	Diffuse midline glioma, H3K27M mutant	8	11	No	No	1,102	Yes	No	3 T	3 mm
30	M	AWD	73	B	Yes	Diffuse midline glioma, H3K27M mutant	5	15	No	No	1,736	Yes	No	3 T	3 mm
31	F	DOD	79	B	Yes	Diffuse midline glioma, H3K27M mutant	3	15	No	No	1,575	No	No	3 T	3 mm
32	M	DOD	65	B	Yes	Diffuse midline glioma, H3K27M mutant	6	13	No	No	2,200	Yes	No	3 T	3 mm
33	F	AWD	111	B	Yes	Diffuse midline glioma, H3K27M mutant	6	13	No	No	1,519	Yes	No	3 T	3 mm
34	F	AWD	30	B	Yes	Diffuse midline glioma, H3K27M mutant	18	18	No	No	1,980	No	No	3 T	3 mm
35	M	DOD	72	B	Yes	Diffuse midline glioma, H3K27M mutant	7	8	No	No	1,833	No	No	3 T	3 mm

*ID, patient number; sex, male (M) or female (F); Status, patient outcome including alive with disease (AWD), dead of disease (DOD) or lost at follow-up (LAF); Age at diagnosis, age at diagnosis expressed in months; Treatment, patient first line of treatment with radiotherapy and Temozolomide (A) or with radiotherapy, Nimotuzumab and Vinorelbine (B); Available histology, available (yes) or non-available (no) histological diagnosis, according to the WHO 2016 classification; Histological diagnosis, diffuse midline glioma, H3K27M mutant, according to the WHO 2016 or n/a, not available; Time to progression, the length of time from the date of diagnosis of DIPG until the radiological and/or clinical progression of the disease, expressed in months; Overall survival, overall survival expressed in months; CN V diagnosis, direct cranial nerve V involvement at diagnosis present (yes) or absent (no); CN V follow-up, direct cranial nerve V involvement at follow-up present (yes) or absent (no); Tumor size, tumor size expressed in mm^2^; Ring enhancement, ring enhancement present (yes) or absent (no) at diagnosis; Dissemination at follow-up, distant localization of the tumor present (yes) or absent (no) on the follow-up scans; Scanner, magnet strength, expressed as 3 Tesla (3 T) or 1.5 Tesla (1.5 T); Slice thickness, slice thickness of the sequences contained in the imaging protocol, respectively 3 millimeters (3 mm) or 4 millimeters(4 mm)*.

Ring enhancement was present in nine patients (3 M; 31% of the cohort). Median tumor size at diagnosis was 1,736 mm^2^ (range 850–2,552 mm^2^).

**Figure 2:**
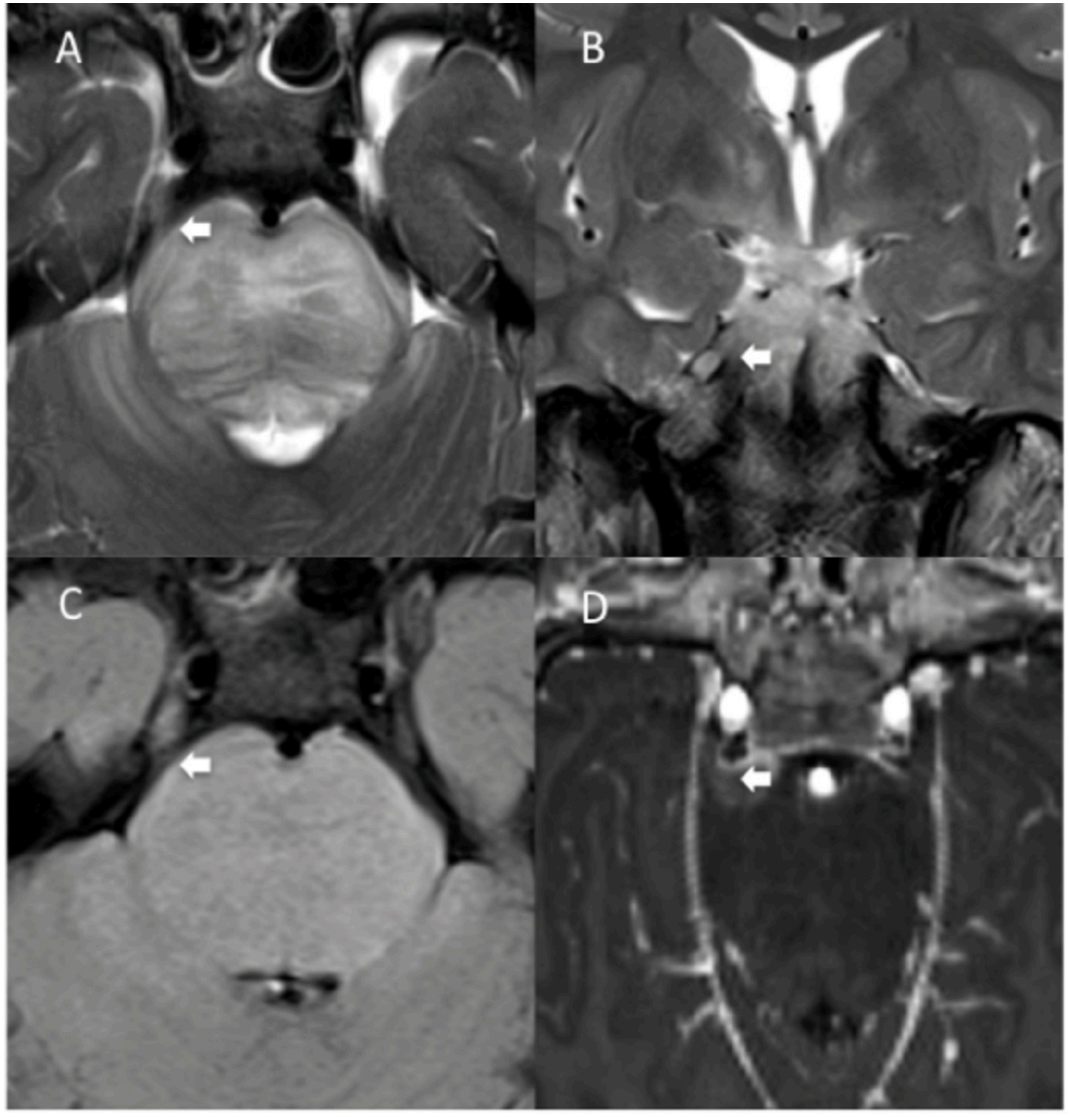
Another MRI diagnosis of direct involvement of CN V by DIPG in a 7-years-old boy. TSE T2 axial **(A)** and coronal **(B)**, axial FLAIR **(C)**, and T1 post-contrast para-axial **(D)** reformatted MRI images show thickening and abnormal signal intensity involving the root entry zone and the cisternal course of the right V cranial nerve, contiguous with the tumor, (**A–C**, white arrows). Findings are consistent with direct right V cranial nerve involvement by the tumor. There is associated contrast enhancement (**D**, white arrow).

Direct trigeminal nerve involvement at diagnosis was found in four patients (3 M, 1 F, 13.8% of the cohort, Figure 2). Two of them (50%) did not show contrast enhancement. Median age at diagnosis in the subgroup with CN V involvement was 4.8 years (range 3–5.6).

**Figure 3:**
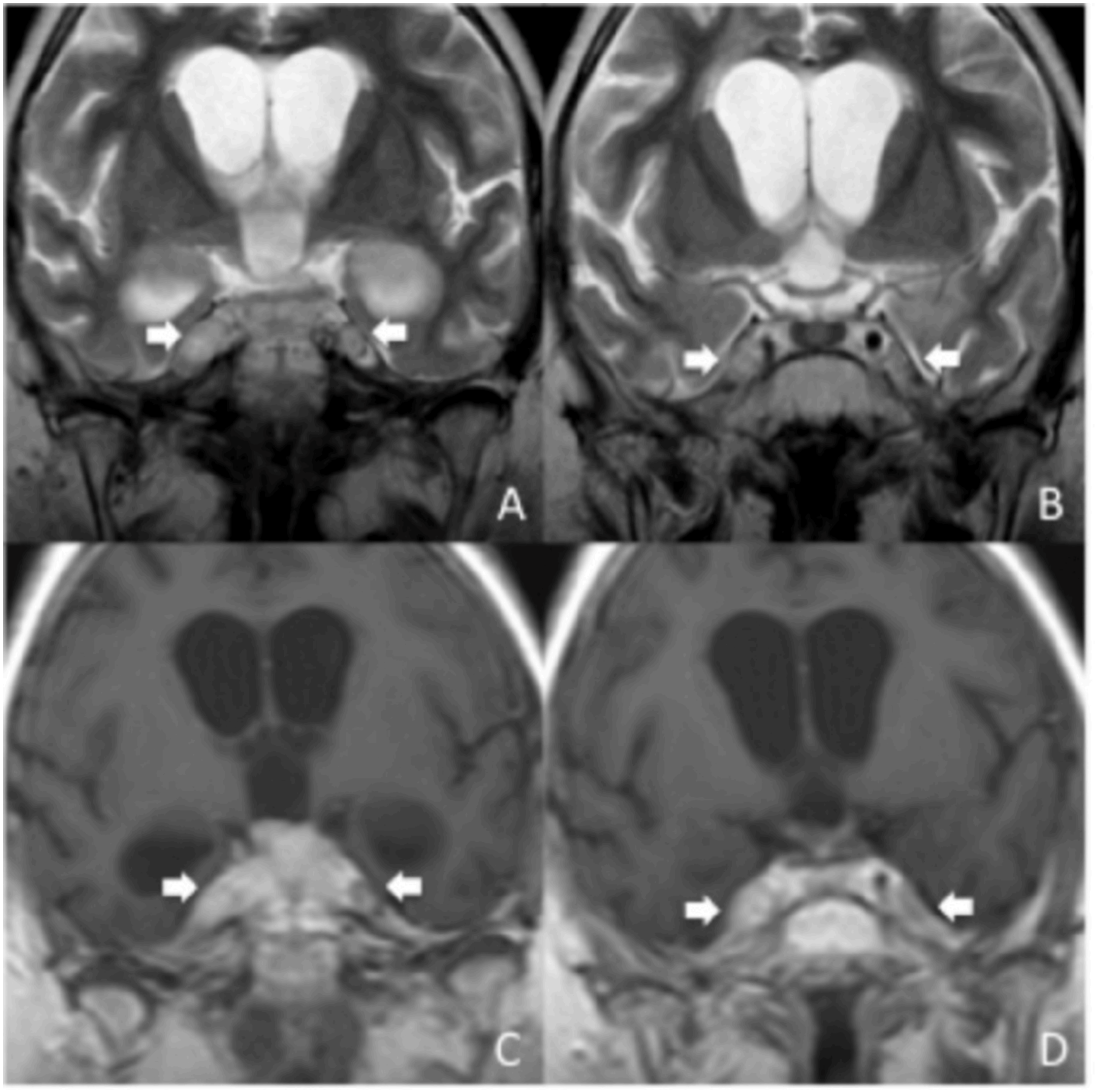
MRI findings of V cranial nerve involvement at follow-up in a 4-years-old girl previously diagnosed with DIPG. Coronal TSE T2 **(A,B)** and post-contrast coronal T1 **(C,D)** images show extensive thickening of both V cranial nerves and abnormal bulging of the Meckel caves, consistent with direct infiltration by the tumor (**A–D**, white arrows). There is associated contrast-enhancement (**C,D**, white arrows).

In two cases (50%), direct involvement of CN V was also confirmed at follow-up. In one of these children trigeminal nerve involvement was bilateral (Figure 3).

Dissemination at follow-up was observed in five patients (17.2%) of the general cohort, two of them belonging to the direct CN V involvement group. At follow-up, one of the two patients with involvement of CN V (50%) also presented dissemination (50%).

Median OS in our cohort was 11 months (range 3–23 months). Median TTP was 7 months (range 2–18 months).

**Figure 4:**
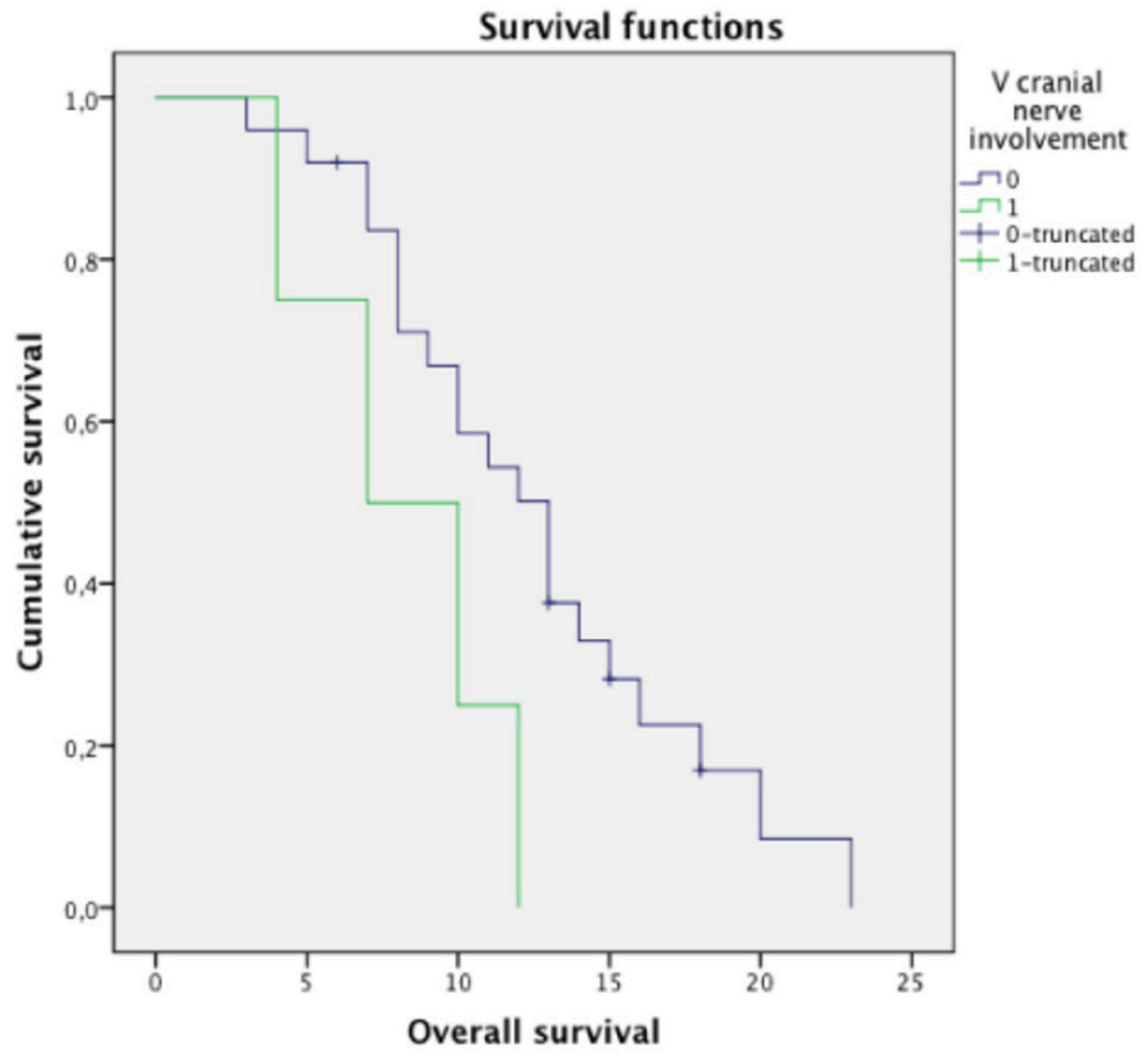
Survival analysis with Kaplan-Meier curves for patients in our cohort presenting with V cranial nerve involvement at diagnosis. Patients with direct involvement of CN V (label = “1,” green line, median OS: 7 months, 95% CI 1.1–12.9 months) presented significantly lower overall survival (OS) compared to the group without involvement of CN V (label = “0,” violet line, median OS: 13 months, 95% CI 10.2–15.7; log rank test: *p* < 0.049). The other parameters (not shown) were not associated with significant differences in OS (*p* = NS).

Log-rank test revealed significant differences in OS between the group with direct involvement of CN V (median OS: 7 months, 95% CI 1.1–12.9 months) and the group without (median OS: 13 months, 95% CI 10.2–15.7; *p* < 0.049, Figure 4). No significant differences in OS were found in relation to ring enhancement, tumor size, treatment protocol, age, and sex (*p* = NS).

**Figure 5:**
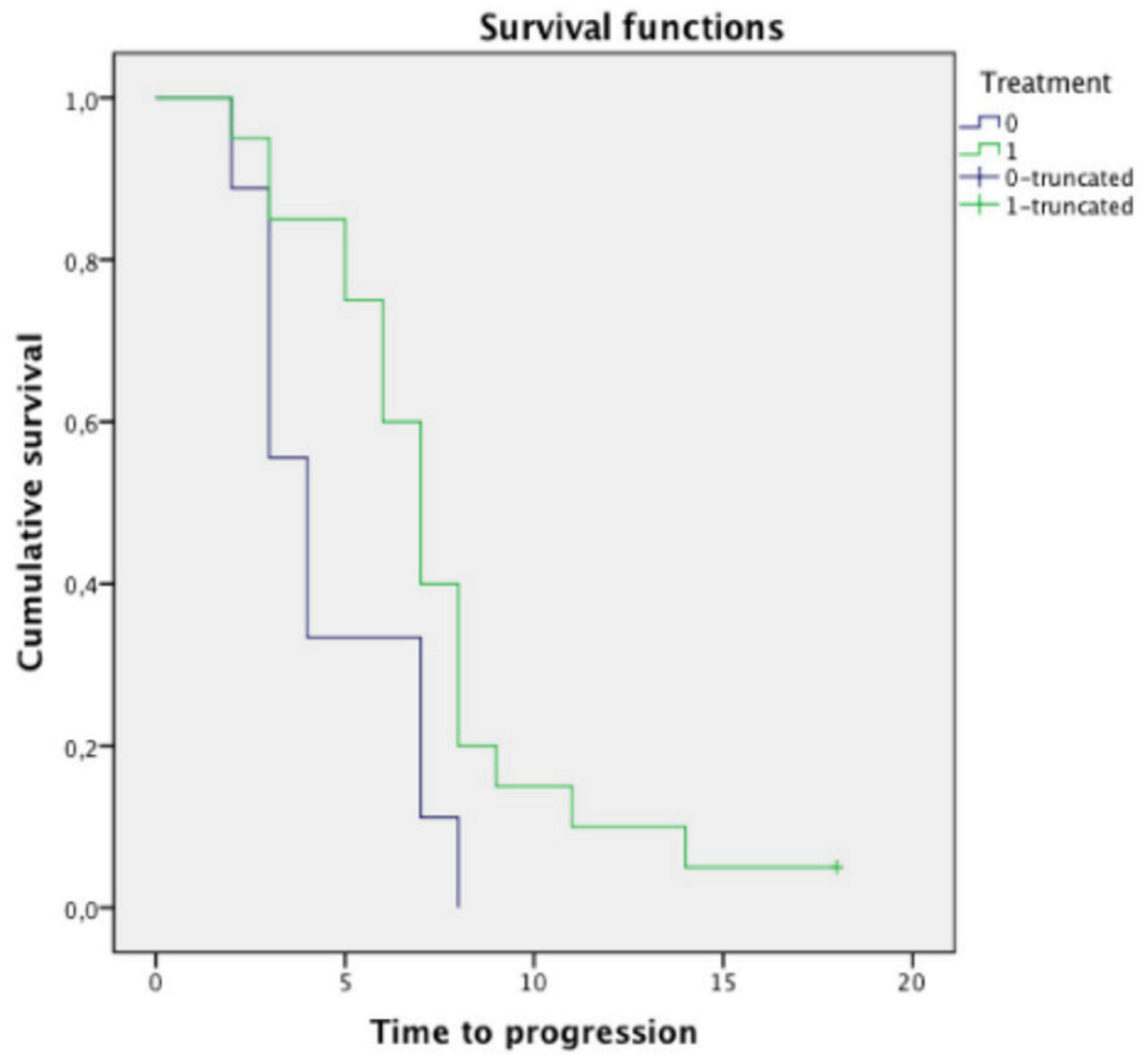
Survival analysis with Kaplan-Meier curves for the type of treatment administered to the patients in our cohort. Patients treated with radiotherapy, Nimotuzumab and Vinorelbine (label = “1,” green line, median TTP: 7 months, 95% CI 5.9–8.1 months) presented significantly higher time to progression (TTP) than patients treated with radiotherapy and Themozolomide (label = “0,” violet line, median TTP: 4 months, 95% CI 2.6–5.3 months, log rank test: *p* < 0.027). The other parameters (not shown) were not associated with significant differences in TTP (*p* = NS).

Sex, age at diagnosis, tumor size at diagnosis, ring enhancement and involvement of CN V did not significantly affect TTP (*p* = NS). Significant differences in TTP were found for different treatment protocols (median TTP: 4 months, 95% CI 2.6–5.3 months for radiotherapy and Temozolomide vs. 7 months, 95% CI 5.9–8.1 for radiotherapy, Nimotuzumab and Vinorelbine, respectively, *p* < 0.027, Figure 5). No significant correlation was found between direct involvement of CN V at diagnosis with magnet strength (*p* = NS) and slice thickness (*p* = NS).

A trend toward a positive correlation was found between direct involvement of CN V at diagnosis and dissemination of disease at follow-up (*r* = 0.347; *p* < 0.065).

## Discussion

In this study we investigated the MRI finding of direct involvement of CN V at diagnosis as a potential biomarker for short-term progression in patients with DIPG. CN V involvement was significantly associated to a shorter OS (Figure 4).

To our best knowledge, there are no studies in the literature specifically focusing on the direct involvement of CN V in DIPG. The reasons for this may be mainly due to the difficulty to objectively assess the direct nerve involvement and the limited available information about the clinical impact of such finding. Due to their anatomical location, DIPGs present a very high probability of direct cranial nerve infiltration. From a clinical standpoint, in these patients cranial nerve palsies are a common manifestation of the disease. However, clinical examination may not be predictive of direct cranial nerve extension of the tumor. Therefore, radiological evaluation could prove valuable were cranial nerve involvement a clinically significant variable in children with DIPG.

We hypothesized that direct involvement of CN V would be more frequent in tumors with a higher propensity to infiltrate and, possibly, to disseminate at follow-up. Since the majority of DIPG show local progression, being able to identify a subgroup of patients with a higher dissemination risk at follow-up may lead to different tailored treatment strategies, i.e., craniospinal radiation.

In our study, patients with direct CN V involvement at diagnosis showed a shorter sOS (7 vs. 13 months, *p* < 0.049) and a higher rate of tumor dissemination at follow-up (*r* = 0.347, *p* < 0.065). Although the small number of patients represents a considerable limit to our analysis, we believe these promising results are worthy of further investigation.

In principle, it is possible that other cranial nerves, such as the VII–VIII, could be infiltrated in patients with DIPG. It has to be considered, though, that due to the characteristics of DIPG it may be difficult to distinguish between nerve involvement and tumor bulging at that level. Therefore, we decided to focus only on the involvement of CN V, where the finding was deemed unequivocal. Our findings (four patients with involvement of CN V, 13.7% of our cohort) suggest that direct involvement of CN V may not be so rare in patients with DIPG (Figures 1–3).

Since CN V is a small structure (the nerve has a caliber of <3 millimeters), spatial resolution of the diagnostic images is important. For this reason, it is possible that magnet strength and/or the slice thickness may influence the detection rate of CN V involvement, and that high-resolution images obtained from 3 T magnets could allow higher detection rates. In our cohort, however, we found no significant correlation between the diagnosis of CN V involvement and magnet strength (*r* = −0.201, *p* = NS) or slice thickness (*r* = −0.201, *p* = NS).

Follow-up scans in our cohort revealed direct involvement of CN V in two of four cases (Table 2). Interestingly, one of the two patients with CN V involvement at follow-up also presented leptomeningeal spread of the disease (50%). Although our population is small, these findings seem consistent with our hypothesis that CN V involvement is more frequent in tumors with a higher propensity to disseminate at follow-up. However, when assessing the predictive value of direct involvement of CN V, different factors should be taken into account, mainly therapy-related tumor changes, and the low number of observed patients with involvement of CN V, which limited further analyses.

Other MRI parameters have been tested for survival prediction (9–14, 17). Among different MRI and clinical features, we analyzed if tumor size, ring enhancement, age, and sex influenced OS in our cohort. Utility of calculating tumor size was debated. Some authors have emphasized the relevance of inter-observer variability (27) and stressed the importance of reproducibility of the measures and single reader measurement of tumor size on DIPG scans. In our cohort, greater tumor size at diagnosis did not modify overall survival, which is consistent with the findings in the literature (15, 16). Some authors have also tested and found a discrepancy between longitudinal volumetric measures and metabolic evolution of the tumor measured by spectroscopy, the latter being better suited to response assessment strategies (28). Ring contrast enhancement has shown a negative correlation with OS in a large retrospective study (17). In our cohort, a trend toward shorter OS for patients without ring enhancement was observed (*p* < 0.78). The reasons behind this result may be due to our small sample size. In the same study age <3 years was correlated to a longer OS (17). Since in our cohort there were only two patients <3 years, we decided to stratify age to the median (5.7 years). As such, age did not show significant correlations with OS. Sex did not show significant correlations with OS, which was also consistent with the findings in literature (17).

Significant differences in TTP were found for patient treatment (median TTP: 4 months, 95% CI 2.6–5.3 months for radiotherapy and Temozolomide vs. 7 months, 95% CI 5.9–8.1 for radiotherapy, Nimotuzumab and Vinorelbine, *p* < 0.027). This suggests that the new therapeutic regimen lengthened the time to progression of disease, even if it did not change OS. Our findings seem to confirm that treatment with radiotherapy in combination with Nimotuzumab and Vinorelbine represents an interesting therapeutic option (26, 29).

No differences in TTP were found for direct involvement of CN V (*p* = NS). Further research should be performed on the correlation between this finding and the results reported for OS for direct involvement of CN V. Sex, age, tumor size and ring enhancement also did not significantly affect TTP (*p* = NS).

We excluded long-term survivors with an OS of more than 24 months in the statistical analysis. The rationale for our decision is that long-term survivors in our cohort significantly differed from the other patients in terms of heterogeneous clinical, histological and radiological characteristics (Table 1).

More specifically, in the long-term survivor cohort:

- Two of the six patients (PT 16 and 24) are still alive with disease (with a median OS of 68 and 183 months, respectively). They also presented a pattern of progression, which is not typical for the disease (PT 16 progressed after 63 months, and PT 24 never progressed);
- Two of the six patients (PT 8 and 16) did not harbor the H3K27M mutation;
- After review, four of these six patients (PT 8, 16, 24, and 28) had radiological features, consistent with atypical DIPG (well-defined borders and exophytic components).

The small group of long-term survivors probably deserves to be included in a larger multicentric study, in order to identify consistent trends and relevant features (30).

Recent advances in the molecular characterization of DIPG have begun to disclose biological signatures possibly associated to outcome and response to treatment (31). The term DIPG itself is slightly out-dated, as it has been shown that most of these tumors present distinct mutations, most notably the H3K27M. In the 2016 revision of the WHO brain tumor classification, these neoplasms were termed as diffuse midline glioma, H3K27M-mutant (32). Imaging studies are currently directing toward highlighting correlations between the relevant imaging findings and the new molecular findings (33, 34). If involvement of CN V was confirmed to be a predictor of poor survival in DIPG, it could be hypothesized that such finding could also have prognostic significance for the newer entity of the diffuse midline glioma H3K27M mutant. In our cohort, we can report the presence of the H3K27M mutation associated to the involvement of CN V, only for one patient. For the other patients, the biopsy had not been performed and so the mutational status could not be tested.

This study has some limitations. Specifically, we acknowledge the limit of a single institution, retrospective study, employing a low number of patients. Clinical practice has shown that a higher order of collaboration between professionals is required in order to improve our understanding and better progress toward treatment of this disease (30, 35). Another limit of the study was the methodology employed for assessment of direct involvement of CN V, which was made by two neuroradiologists in consensus, even if we tried to mitigate that issue by employing two neuroradiologists from different institutions. Furthermore, for 17 out of 29 patients in our cohort, tissue samples for histological confirmation of the H3K27M mutation were not available. This is because the stereotactic biopsy procedure was introduced in our institution from 2015.

In conclusion, our data suggest that direct involvement of CN V is a surrogate biomarker of poor survival in patients with DIPG, which could be assessed on conventional images. Further studies on larger cohorts, possibly associated with correlation to histological and molecular findings, should be performed to cross validate our results and further test our hypotheses.

## Data Availability

All datasets generated for this study are included in the manuscript and/or the supplementary files.

## Author Contributions

All authors listed have made a substantial, direct and intellectual contribution to the work, and approved it for publication.

### Conflict of Interest Statement

The authors declare that the research was conducted in the absence of any commercial or financial relationships that could be construed as a potential conflict of interest.
